# Comprehensive assessment of scale formation, corrosion, and biological pollution in a cooling water supply system

**DOI:** 10.1016/j.mex.2025.103154

**Published:** 2025-01-09

**Authors:** Olha Biedunkova, Pavlo Kuznietsov, Oleg Pinchuk

**Affiliations:** National University of Water and Environmental Engineering, 33028 Rivne, Ukraine

**Keywords:** Corrosion rate, Scale formation rate, Colony forming unit, Biofilm, Algae cells, Hydrobox, The comprehensive method for assessing the scale formation,corrosion,and biological pollution in CWS of a NPP.

## Abstract

A comprehensive method for the cooling water supply system (CWS) was investigated, which allows for the comprehensive assessment of corrosion, scale formation and biological pollution. Direct and indirect methods were used. Consequently, indirect methods included the calculation of differences in φ and ψ, the Langelier saturation index (LSI), and the Ryznar stability index (RSI), which characterise scale formation and corrosion processes based on the results of the pH, temperature, total dissolved salts, total hardness and total alkalinity measurements were measured using standard methods. Direct methods included bench tests to measure the corrosion rate (V) and scale formation rate (S). Additionally, the colony forming units (CFU) controlling with indication of an yeast and bacteria, the algae cell counts and hydrobox controlling water CWS were carried out. The comprehensive assessment method presented in this article includes adjustments to the existing method to improve its effectiveness, which is used for monitoring of spent fuel storage facilities at a Nuclear Power Plant (NPP).

• Introduced a comprehensive methodology combining direct and indirect monitoring for assessing scale formation, corrosion, and biological pollution in nuclear power plant (NPP) cooling water systems.
• Enhanced monitoring techniques enable the simultaneous assessment of corrosion resistance and scale formation potential, overcoming limitations of traditional methods.
• Incorporated colony-forming unit analysis, algae cell quantification, and hydrobox-based evaluations for a detailed understanding of biofouling.
• Validated methodology offers predictive risk modelling and optimised strategies to ensure reliability and safety in NPP operations.

## Graphical abstract

**Figure:**
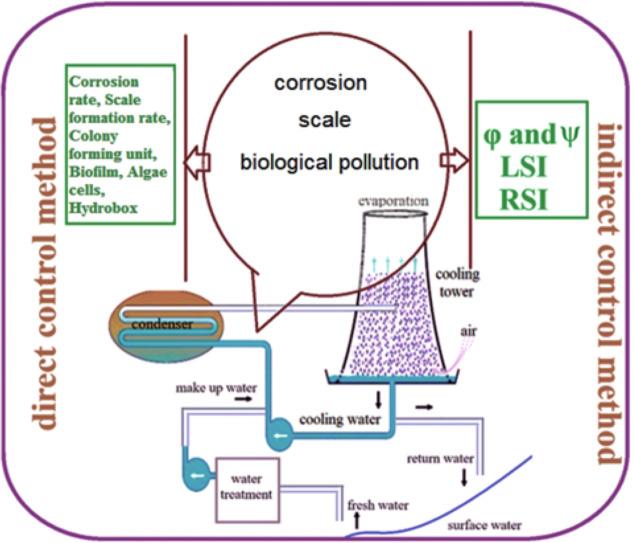

Specifications table

**Table:** 

Subject area:	Energy Engineering and Power Technology.
More specific subject area:	Energy data.
Name of your method:	The comprehensive method for assessing the scale formation, corrosion, and biological pollution in CWS of a NPP.
Name and reference of original method:	N/A.
Resource availability:	Data are reported here and accessible in [1]. Indirect methods, including the calculation of the difference between φ and ψ, the Langelier Saturation Index (LSI) and the Riznar Stability Index (RSI). Bench tests, for direct methods, were carried out according to [2]. The corrosion rate (V) were measured according to [3], the scale formation rate (S) were measured according to [4]. In addition, by the direct method in water was measured colony forming unit (CFU), the number of algae cells, and control with hydrobox was carried out according to [5,6]. All the data were statistically analysed to characterize the distribution and the errors connected to the database built with a definition (min–max), arithmetic mean (M), standard deviation (SD) and using the Pearson correlation analysis with the Pearson correlation coefficient (ρ) [7]. Statistical processing of the research results was carried out using the BioEstar software package (Version 5.3, MLM).

## Background

The scale formation, corrosion, and biological pollution cause technological problems in the operation of the CWS of each power plant. These problems can lead to clogged pipes, reduced heat transfer efficiency, and serious corrosion problems with equipment and pipelines. Additionally, it can result in increased operation and maintenance costs. It is important to address this issue promptly to avoid these potential consequences by providing a comprehensive evaluation of potential scale formation, corrosion, and biological pollution. In an operational practice, the CWS power plant typically uses either direct or indirect methods. However, these methods may not provide a complete understanding of the current processes occurring within the CWS. The motivation behind providing this method is to introduce a comprehensive approach to the assessment of scale formation, corrosion, and biological pollution, which can help to improve the overall performance of the CWS. A number of studies have investigated the corrosivity and scaling potential of water systems using integrated graphical [8,9], statistical [10,11], and index-based methods, as well as artificial neural network modelling [12]. These studies have been conducted in a range of environments, including industrialised regions, agrarian areas, and hydrogeochemical systems, and have yielded valuable insights into water quality. This study introduces a novel integrated methodology for monitoring and controlling key challenges in CWS at nuclear power plants NPPs, focusing on corrosion, scale formation, and biological pollution. Unlike conventional methods that rely on either direct or indirect monitoring, the proposed approach synergistically combines both. Indirect methods, such as LSI and RSI, are augmented with direct bench-test measurements of corrosion rates (V) and scale formation rates (S). Additionally, biological contamination is assessed through colony-forming unit (CFU) counts, algae cell quantification, and hydrobiological evaluations using a hydrobox system. This comprehensive approach enhances process understanding by providing simultaneous evaluations of scaling and corrosion while integrating biological pollution control. Particularly relevant for nuclear engineering, the methodology supports safe and efficient NPP operations by enabling predictive risk modelling and optimizing water treatment strategies. Its potential to improve operational efficiency and environmental sustainability makes it invaluable for NPPs employing recirculating cooling systems. This study also outlines environmental implications, emphasizing the importance of sustainable discharge practices. The findings bridge significant knowledge gaps, offering a robust framework for advanced engineering applications in CWS monitoring where such integrated approaches are not yet adopted. The purpose of the study was to develop and validate a comprehensive method for assessing and mitigating the effects of scale formation, corrosion and biological pollution in the CWS of the NPP, which will help to improve operational reliability and environmental sustainability.

## Method details

**Fig. 1:**
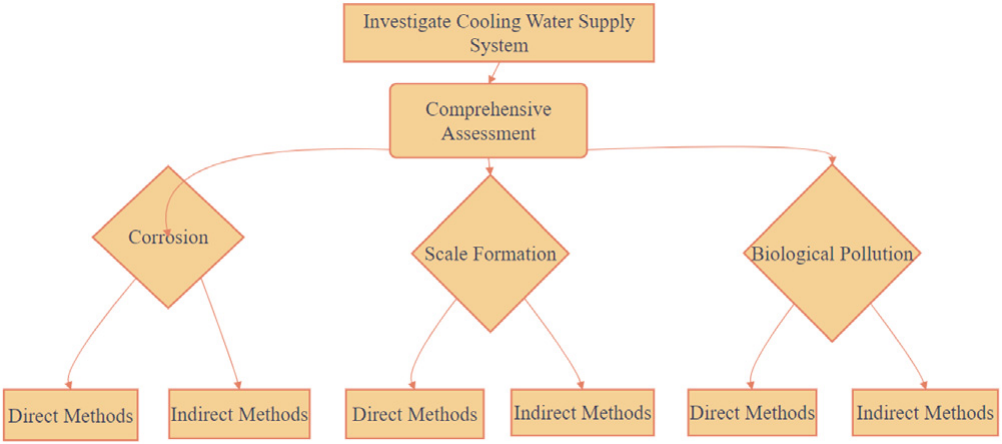
Schematic representation of the use of direct and indirect monitoring methods in this study.

Monitoring of scale formation, corrosion, and biological pollution determination is mandatory in an operation of the CWS NPP [13]. This data assessment enables predictive modelling of risks under varying operating conditions or the development of more efficient water treatment strategies. In addition to technological problems, an operation of a CWS NPP is associated with an environmental impact factor, as the water discharge is directed to water bodies [14]. This requires the implementation of effective methods for controlling the processes occurring in the CWS NPP. The purpose of this study is to apply the comprehensive method for controlling the processes of the scale formation, corrosion and biological pollution in the CWS NPP. It also includes adjustments to the existing methodology used to monitor the CWS, including the use of direct and indirect monitoring methods (Fig. 1), to improve its effectiveness.

**Fig. 2:**
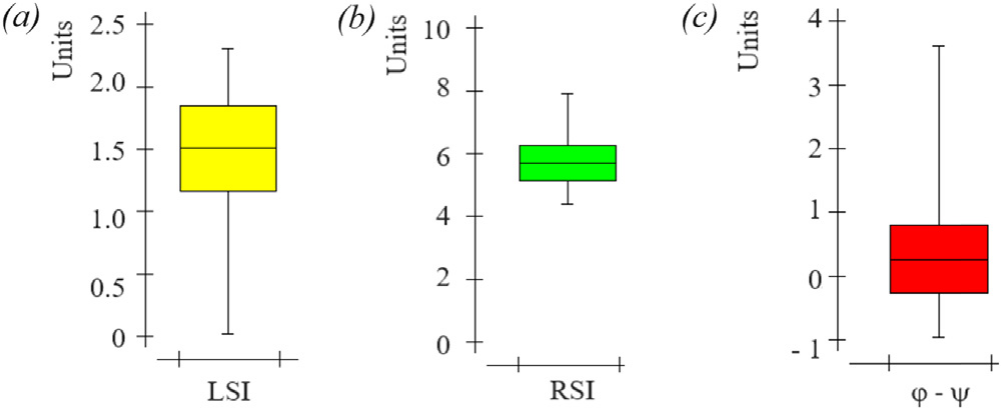
Values of the φ - ψ (a), RSI (b), and LSI (c) for water the CWS RNPP.

The scaling and corrosive potential were determined by the φ - ψ, LSI, and RSI. The following physicochemical parameters were determined according to standard methods described in [1]: the pH, temperature, concentration of dissolved salts, chloride, total hardness, and total alkalinity. The results of the calculations are shown in Fig. 2. Thus, values of the φ - ψ were: min-max −0.91 – 3.61, *M* = 0.245, and SD = ± 0.521; the values of the LSI were: min-max 0.02 – 2.22, *M* = 1.56, and SD = ± 0.381; the values of the RSI were: min-max 4.17 – 7.95, *M* = 5.54, SD = ± 0.58. Moreover, a very strong relationship has been identified (ρ = −0.9635) only between the LSI and RSI. In addition, the interpretations of the indices (φ - ψ, LSI, and RSI) only consider corrosion or scale formation. This is a disadvantage of indirect methods, as they do not allow for the simultaneous monitoring of corrosion resistance and scale formation.

**Fig. 3:**
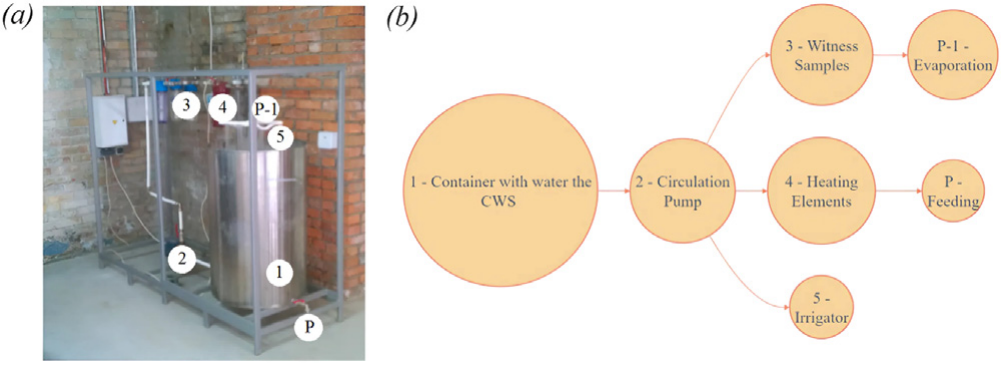
Photo (a) and schematic diagram of the test bench (b).

The test bench (Fig. 3) was designed according to [2] and was used for the direct method of determining the corrosion and scale formation. Placing the samples after the heat exchanger would indeed better replicate the real operational conditions of pipelines that transport heated water, which typically exhibits higher corrosive potential due to elevated temperature, accelerated chemical reactions, and potentially increased turbulence. However, the placement of the witness samples in this study was designed to measure baseline corrosion and scale formation rates under controlled conditions. The test bench setup sought to ensure reproducibility and comparability of results while isolating specific variables influencing corrosion and scale formation. The method is designed to circumvent external variables that could potentially introduce inconsistency, such as transient temperature fluctuations or flow dynamics, which are more challenging to standardise within a laboratory setting. The material (witness samples) of St.20 steels (carbon steel, C content of 0.16–0.24 %) [15], which are defined as analogues of the CWS Rivne Nuclear Power Plant (RNPP) structural material was used. The determination methods and procedures used were based on the regulations of the corrosion resistance of water treatment agents (GB/t 18175–2014) [16]. Three witness samples for measuring the corrosion rate and two heating elements for measuring the scale formation rate were examined simultaneously in the bench tests. The V and S were calculated using the Eqs. (1, 2), respectively.

(1) $$V=K\cdot \left(W_{1}-W_{2}\right)/\left(F\cdot t\cdot g\right)$$

(2) $$S=\left(M_{0}-M_{1}\right)/\left(F\cdot t\right)$$

where W_1_ and W_2_ are the witness samples before and after the test bench (a), respectively; F is the surface area of the witness samples; K is a constant, whose value is 87.6; t is the specimen immersion time; g is the metal density; M_0_ and M_1_ are the mass witness samples before and after the test bench, respectively.

**Fig. 4:**
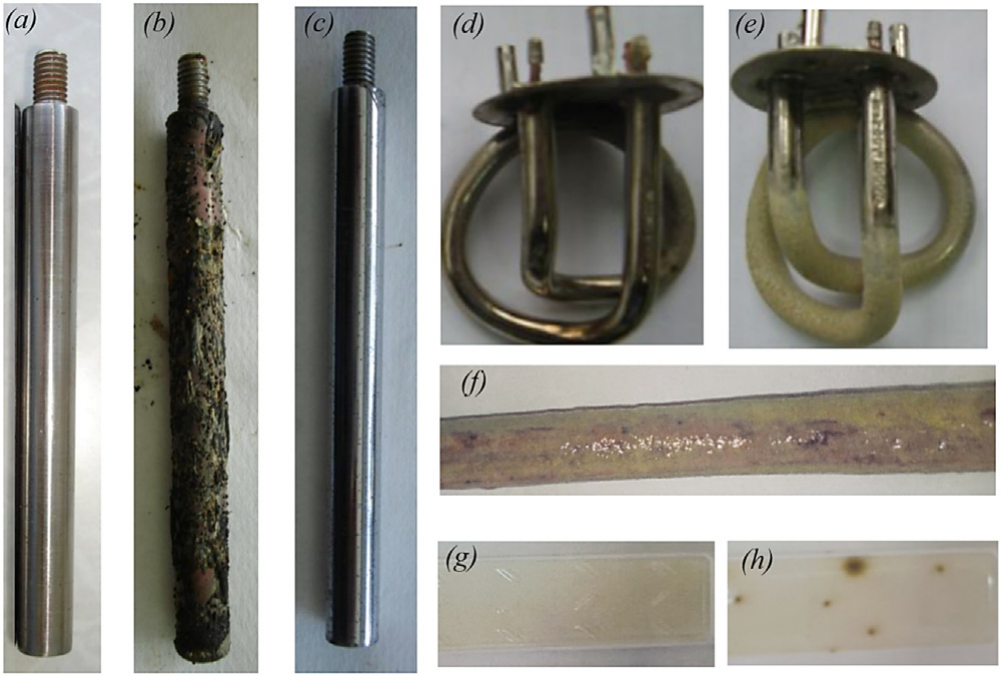
Photos of the witness samples before (a), after (b), and after the removal of deposits were subjected to bench testing (c); the heating elements before (d), and after (e) bench testing; the biological pollution (biofilm) after bench testing (f); and the measurements of the biological pollution before (g), and after (h) bench testing.

**Fig. 5:**
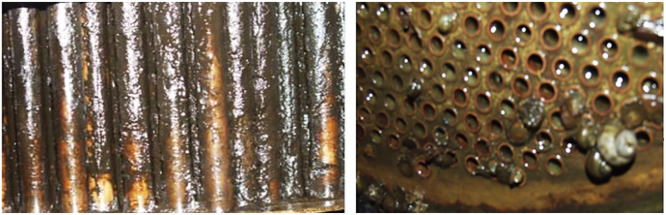
Biofilm on the surface of heat exchangers of the CWS consumers.

After 800 h of the test bench operation, the stability of the corrosion rate is observed. Thus, values of the corrosion rate V were: min-max 0.25 – 0.35 g/(m^2^·h), *M* = 0.305 g/(m^2^·h), SD = ± 0.022 g/(m^2^·h). According to the requirements of the standard [17], the corrosion rate, which characterises the stable corrosion state of a material, should not exceed 0.5 g/(m^2^·h). Photos of witness samples before and after bench testing are shown in Fig. 4a. After bench testing, the presence of deposits was noted on the surface of the witness samples Fig. 4b. Moreover, no localised corrosion damage was observed on the surface of the witness samples after removal of the deposits Fig. 4c. The rate of scale formation was also determined in bench tests on heating elements (Fig. 4d,e) that provided a maximum water temperature of 45 °C in the CWS RNPP. Thus, values of the rate of scale formation S were: min-max 0.52 – 0.75 g/(m^2^·h), *M* = 0.63 g/(m^2^·h), SD = ± 0.08 g/(m^2^·h). The biological pollution (biofilm) was detected on the surface of the witness samples after bench testing (Fig. 4f). To measure the biological pollution of the water the CWS at the end of the bench tests, the test system was used to determine bacteria (Agar environment, Fig. 4g,h showed moderate contamination of the water the CWS by bacteria corresponding. The contamination levels obtained for the water CWS indicate the need to introduce а biocidal treatment [6,18] to minimise biological pollution processes. It is also important to note that there is also a biofilm on the internal surfaces of the ССS equipment (Fig. 5), which requires the implementation of methods to prevent biological pollution, in particular а biocidal treatment.

**Fig. 6:**
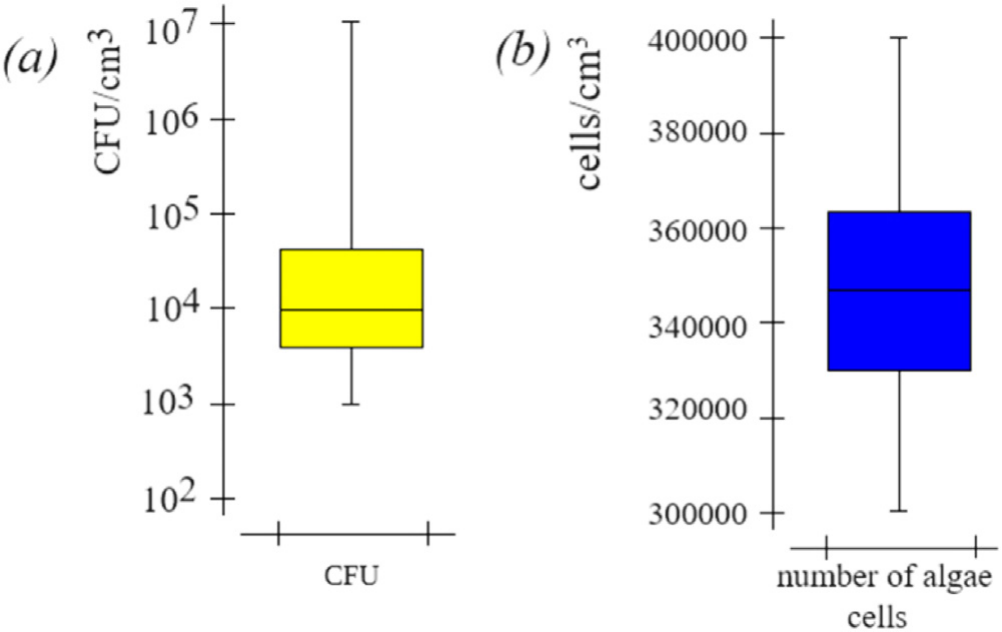
Values of the CFU (a), and the number of algae cells (b) for water the CWS RNPP.

The colony forming unit (CFU) and direct cell count determination of the water samples. To measure the CFU of the cooling water CWS the Orion test system was used. The CFU values of the cooling water CWS were highest in summer and autumn and lowest in winter. Typically, the CFU values range between 1 and 100 million CFU/cm^3^ and the composition and number of biological pollution [19]. The CWS water CFU values varied in the min-max range of 10^3^ - 10^7^ CFU/cm^3^, *M* = 10^4^ CFU/cm^3^, and SD = ± 10^3^ CFU/cm^3^ (Fig. 6a). Changes in CFU values in CWS water depend on the source of the water supply, seasonality and water-chemical mode.

**Fig. 7:**
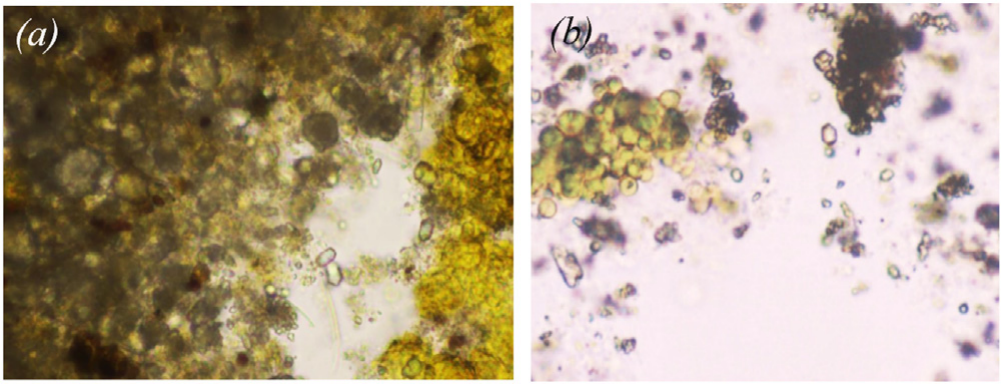
Microscopy during the calculation of the number of algae cells in the exposure chamber for water the CWS RNPP: yellow-green algae Tribonema (a) and green algae Ladophora Glomerata, Chlorophyta (b).

However, yeast and bacteria are not the only representatives of biological pollution and biofilms on the surfaces of CWS. The operation of the CWS equipment is also seriously affected by algae, especially when phosphorus-containing compounds are used as a corrective agent for descaling [5]. This requires the introduction of systematic control of biological pollution of CWS water due to the presence of algae. For the control in this study, a direct method of counting the number of algae cells in the exposure chamber was used [6]. Additionally, algae were identified in the water samples via microscopy, and yellow of yellow-green algae and green algae were detected (Fig. 7). The CWS water the number of algae cells values varied in the min-max range of 300,000 - 400,000 cells/cm^3^, *M* = 348,000 cells/cm^3^, SD = ± 20,000 cells/cm^3^ (Fig. 6b).

**Fig. 8:**
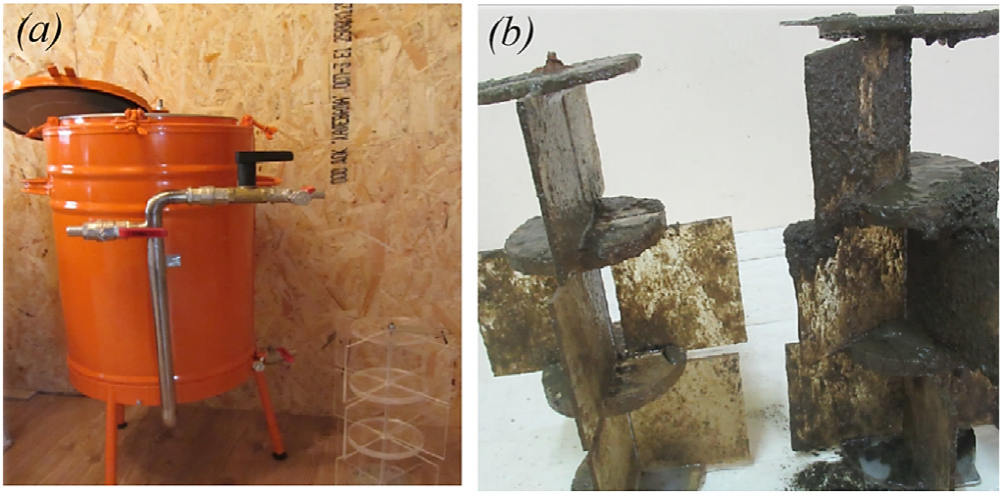
The hydrobox for the control of biological pollution of water the CWS: external appearance (a), internal tab (b).

Furthermore, invertebrates (molluscs, snails, etc.) also create serious biological exploitation, they can clog the internal surfaces and pipeline of the CWS equipment [20]. This study involves monitoring with the hydrobox (Fig. 8), which is a cylindrical container with a set of plates (plexiglass) that is connected to the water flow of the CWS. The hydrobionts are retained in the hydrobox, and after a certain period of time, the hydrobox is inspected to identify the hydrobionts and measure their number by counting.

In summary, there is an urgent need to ensure comprehensive control of scale formation [[21], [22]–23], corrosion [1,24,25], and biological pollution [26,27] in the CWS. Thus, the results obtained by direct and indirect methods complement each other, and the informational content of control monitoring is increased.

## Method validation

**Fig. 9:**
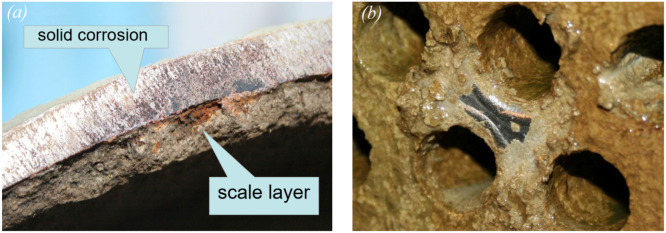
The internal surfaces of consumer equipment of the CWS: the corrosion and scale formation processes (a) and localised corrosion under scale (b).

Samples were collected and subjected to bench tests at the RNPP, Ukraine; analyses were performed at the Measurement Laboratory, RNPP, Varash, Rivne Region, Ukraine. Monitoring data processing was performed by the National University of Water and Environmental Engineering, Rivne, Ukraine. The study results are presented in this article. Notably, this comprehensive method allows us to identify the corrosion and scale formation processes that occur simultaneously in the equipment of the power plant CWS (Fig. 9).

The objectives of this method were: to combine direct and indirect methods for simultaneous assessment of corrosion and scale formation processes at CWS; - to implement and validate biological monitoring methods, including CFU counting, algal cell quantification and hydroxide-based assessments, to monitor biological fouling at CWS; - improve existing methodologies to account for the interaction of chemical and biological factors in real-world conditions at the NPP; - expand predictive modelling capabilities to assess the risks of scale formation, corrosion and biological risks under variable operating conditions; - support the sustainable operation of wastewater treatment plants by providing practical recommendations for mitigating environmental impacts and optimising water treatment.

The control of the scale formation, corrosion, and biological pollution in the CWS by indirect methods described in this article is carried out at a NPP according of the standard [17]. However, it does not allow identification of the scale formation and corrosion processes to [1] that occur simultaneously. Instead, direct methods are known, such as bench tests of the corrosion and scale formation processes with the determination of V and S. This study proposes the adjustments made to an existing method to improve its effectiveness, which is used to monitor the CWS at a NPP according to [20]. In particular, the indirect methods of calculating φ - ψ, LSI, and RSI are supplemented with direct methods with measurements of V and S. This will allow supplementing information on corrosion and scale formation processes in the CWS. Moreover, the addition of biological pollution control by CFU, of the number of algae cells in the exposure chamber and the hydrobox will also allow identifying the identification of undesirable processes that affect the operation of the CWS.

This study presents a comprehensive methodology for assessing and monitoring scale formation, corrosion, and biological pollution in CWS of NPP. By integrating indirect methods, such as LSI and RSI, with direct measurements of corrosion and scale formation rates, the proposed approach addresses critical limitations of conventional monitoring techniques. Furthermore, the incorporation of advanced biological pollution control measures, including CFU analysis, algae cell quantification, and hydrobiological evaluations, enhances the capability to detect and mitigate undesirable processes affecting CWS performance. The findings demonstrate that this integrated method provides a more holistic understanding of the interactions between chemical and biological factors in CWS, ensuring more reliable and efficient system operation. The methodology's applicability extends to NPPs and other industrial facilities with similar systems, offering a robust framework for predictive risk management and sustainable water treatment strategies.

## Limitations

This method can be applied to any power plant with a CCS and has no restrictions. To control the corrosion rate, when using the direct control method, an analogue of the structural materials should be selected as witness samples of a particular a NPP.

## CRediT authorship contribution statement

**Olha Biedunkova:** Supervision, Software, Validation, Writing – review & editing. **Pavlo Kuznietsov:** Software, Data curation, Writing – original draft, Visualization. **Oleg Pinchuk:** Conceptualization, Methodology, Investigation.

## Declaration of competing interest

The author declares that they have no known competing financial interests or personal relationships that could have appeared to influence the work reported in this paper.

## Data Availability

https://doi.org/10.17632/3pd3x2wcmx.1

## References

[1] Kuznietsov P. (2024). Evaluation of the scaling and corrosive potential of the cooling water supply system of a nuclear power plant based on the physicochemical control dataset. Data Brief.

[2] Kuznietsov P.M., Biedunkova O.O., Yaroshchuk O.V. (2023). Experimental study of transformation of carbonate system components cooling water of Rivne Nuclear Power Plant during water treatment by liming. Problem At. Sci. Technol..

[3] Hsieh M.K., Dzombak D.A., Vidic R.D. (2010). Bridging gravimetric and electrochemical approaches to determine the corrosion rate of metals and metal alloys in cooling systems: bench scale evaluation method. Ind. Eng. Chem. Res..

[4] Zhao S., Jing Y., Liu T., Zhao W., Li F. (2024). Corrosion behavior and mechanism of carbon steel in industrial circulating cooling water system operated by electrochemical descaling technology. J. Clean. Prod..

[5] Kuznietsov P.М., Biedunkova O.О., Yaroshchuk O.V., Рryshchepa A.M. (2024). Optimization of the anti-scale corrective treatment of water by organic phosphonate. Sci. Innov..

[6] Moheimani N.R., Borowitzka M.A., Isdepsky A., Sing S.F. (2013). Standard methods for measuring growth of algae and their composition. Algae for biofuels and energy. Dev. Appl. Phycol..

[7] Schober P., Boer C., Schwarte L.A. (2018). Correlation coefficients: appropriate use and interpretation. Anesth. Analg..

[8] Boualem B., Egbueri J.C. (2024). Graphical, statistical and index-based techniques integrated for identifying the hydrochemical fingerprints and groundwater quality of In Salah, Algerian Sahara. Environ. Geochem. Health.

[9] Singh G., Wani O.A., Egbueri J.C. (2023). Seasonal variation of the quality of groundwater resources for human consumption and industrial purposes in the central plain zone of Punjab, India. Environ. Monit. Assess..

[10] Omeka M.E., Egbueri J.C., Unigwe C.O. (2022). Investigating the hydrogeochemistry, corrosivity and scaling tendencies of groundwater in an agrarian area (Nigeria) using graphical, indexical and statistical modelling. Arab. J. Geosci..

[11] Alum O.L., Abugu H.O., Onwujiogu V.C. (2023). Characterization of the Hydrochemistry, Scaling and Corrosivity Tendencies, and Irrigation Suitability of the Water of the Rivers Karawa and Iyiaji. Sustainability.

[12] Egbueri J.C., Unigwe C.O., Agbasi J.C. (2023). Indexical and artificial neural network modeling of the quality, corrosiveness, and encrustation potential of groundwater in industrialized metropolises, Southeast Nigeria. Environ. Dev. Sustain..

[13] Kuznietsov P., Biedunkova O. (2024). Assessment of the impact of organic matter discharge from a nuclear power plant with a recirculating cooling water system. Water Air Soil Pollut..

[14] Biedunkova O., Kuznietsov P., Gandziura V. (2024). Behaviour of dissolved inorganic salts in the cooling water of a nuclear power plant open recirculation system and formation of water discharge. R. Soc. Open Sci..

[15] Information on http://online.budstandart.com/ru/catalog/doc-page?id_doc=52150 (Standart in Ukraine DSTU GOST 17378:2003).

[16] Chen Y.M., Sun C.X., Cheng Y.S., Zhang M., Chen M. (2014). Study on corrosion inhibition of carbon steel, copper and stainless steel by the polyaspartic acid compound. Appl. Chem. Ind..

[17] Information on https://online.budstandart.com/ru/catalog/doc-page.html?id_doc=109230 (Standart in Ukraine SOU NAEK 067:2023)

[18] Trach Y., Trach R., Kuznietsov A., Pryshchepa O., Biedunkova A., Kiersnowska I. (2024). Statnyk predicting the influence of ammonium toxicity levels in water using fuzzy logic and ANN models. Sustainability.

[19] Kéki Z., Makk J., Barkács K. (2019). Critical point analysis and biocide treatment in a microbiologically contaminated water purification system of a power plant. SN Appl. Sci..

[20] Lin H., Zhang S., Cao R., Yu S., Bai W., Zhang R., Yang J., Dai L., Chen, Zhang Y., Xu H., Liu K., Zhang X. (2024). A review on the risk, prevention and control of cooling water intake blockage in coastal nuclear power plants. Nucl. Eng. Technol..

[21] Du Plessis G.E., Liebenberg L., Mathews E.H., Du Plessis J.N. (2013). A versatile energy management system for large integrated cooling systems. Energy Convers. Manag..

[22] Biedunkova O., Kuznietsov P., Korbutiak V. (2024). Evaluation of return cooling water reuse in the wet cooled power plant to minimise the impact of water intake and drainage. Sustain. Chem. Environ..

[23] Jia L., Wei S., Liu J. (2021). A review of optimization approaches for controlling water-cooled central cooling systems. Build. Environ..

[24] Zhao S., Jing Y., Liu T., Zhao W., Li F. (2024). Corrosion behavior and mechanism of carbon steel in industrial circulating cooling water system operated by electrochemical descaling technology. J. Clean. Prod..

[25] Harrington C., Baron-Wiechec A., Burrows R. (2019). Chemistry and corrosion research and development for the water cooling circuits of European DEMO. Fusion Eng. Des..

[26] Kuznietsov P.M., Biedunkova; O.O. (2024). Multivariate regression studies for the investigation of the COD, BOD, and TOC concentrations in the water of the Styr River within the zone of influence of the power plant discharge. Water Pract. Technol..

[27] Gang W., Wang S., Xiao F., Gao D.C. (2016). District cooling systems: technology integration, system optimization, challenges and opportunities for applications. Renew. Sustain. Energy Rev..

